# Moral trauma caused by COVID-19 - An ethical debate

**DOI:** 10.12669/pjms.37.3.4295

**Published:** 2021

**Authors:** Shabih H. Zaidi, Shaukat Ali Jawaid

**Affiliations:** 1 Shabih Zaidi, NHS- UK, SIVU E-mail: shabih514@gmail.com; 2 Shaukat Ali Jawaid Chief Editor, Pakistan Journal of Medical Sciences, Karachi - Pakistan. E-mail: pjms@pjms.com.pk

COVID-19 has introduced a couple of new terminologies to the medical profession. One of them is Moral Trauma which will be discuss here. And the second equally fascinating term introduced in the UK only recently is “Vaccine Nationalism’. Of course we are quite familiar with the term nationalism in political sense but not in terms of medical sciences. So it is somewhat of a novice.

In late January this year, a battle broke out between several European countries and the UK on the supply of AstraZeneca Vaccine. Politicians on both sides continued to hide their real motives, as they always do under the guise of needs and necessities, roles and responsibilities, sincerity to their people, and what they call the Vaccine Nationalism. It basically implies that the countries manufacturing the gold dust aka corona vaccine may keep it to themselves. The opponents say that the manufacturing countries in the EU are morally obliged to share their produce with their neighbouring countries such as the UK.

Plentiful supplies of vaccines are available in the UK. By 15th February the NHS had vaccinated nearly all above 80 years of age and claimed to have reached their target of vaccinating about a quarter of its population. Fifteen million people have so far been vaccinated. But still a vast supply of vaccines is needed to reach that target and to vaccinate the nearly all adult and otherwise vulnerable population. And that is why the battle. It is the same old question of supply and demand which has brought in so much bitterness amongst the politicians on both sides of the English Channel.

Well, there might be some factual reasoning behind the arguments but if that is the gold standard then how about the appeal of the WHO Director General pleading for equitable distribution of vaccines to the developing and underdeveloped nations.

Pakistan has started its vaccination programme with the Healthcare workers which are the frontline warriors vaccinating them with the Chinese donation of Covid vaccine (CanSino BIO vaccine). This vaccine had Phase-III trial in Paksitan at five centers the biggest one being the University of Health Sciences Lahore under the supervision of Prof. Javed Akram VC UHS who was the principal investigator here and they enrolled the maximum number of volunteers over ten thousand. Initial results released shows that it has 65.7% efficacy besides being 99.98% effective in preventing severe disease.^1^UHS has already started trial of another Chinese Covid vaccine (ZF2001 Vaccine). Some private medical institutions have also announced offering imported Covid vaccines from Russia (Sputnik V). Its price in Pakistan is almost four times the price at which this Russian vaccine is available in India.

The Drug Regulatory Authority of Pakistan (DRAP) has so far granted registration to quite a few vaccines and fixed their price. In the days to come some more vaccines may be registered which might bring the cost down. Registration for administration of Covid vaccine to elderly people over sixty years of age with comorbidities who are at greater risk has also started by the government. Arrangements for vaccination made by the provincial governments of Punjab and Sindh are excellent. Walk in vaccination facility is available for people over seventy years of age while those above sixty years are now also being registered. Knowing our culture well, one just wonder how the authorities would prioritize the immunization programme. Prioritization demands transparency, equity, fairness and accountability. These are its pillars.

Triage and prioritization are common practices in the NHS in the UK which are routinely applied in day to day work. However it was emphatically witnessed during the pandemic in the NHS. The discipline shown by the public, despite the lack of trust in the politicians, was a lesson for lesser mortals. Strict criteria was observed right across the nation. People waited for their turn to be vaccinated. It was a fair, simple and routine process that we all went through for our first dose. No rush, no favoritism, no abuse of authority or power. And now everyone waits patiently for the second call.

In Pakistan, time alone will determine if the initial supplies of vaccines are distributed among the most vulnerable people according to a plan, or given first and foremost to those in power or their relatives. If such a practice is witnessed then a major burden should weigh upon their conscience. It is called Moral trauma.

We saw it in the UK and the USA when many health professionals bore the moral trauma upon their conscience so intensely that some have either resigned or simply accepted their failure, inability to help the dying humanity due to the lack of supplies. It was not their failure, only of the State, as it is the State that is responsible for health care.

Health and wellbeing is an individual’s responsibility as one must take good personal care with a balanced diet, exercise, clean water and fresh air. But, an individual does not own or run a hospital or a healthcare facility, nor make any policy decisions regarding climate. It is the responsibility of the state. Charities, NGOs and the private sector play a major role in supplementing many such services as we see in many parts of Asia and Africa. Pakistan excels in charitable services to humanity.

By definition, health is not just freedom from disease, illness, impairments or disabilities, but a state of holistic wellbeing of individuals who collectively form a community. So, the relationship between a society and the State is intricate just like a delicately woven tapestry. It goes back to the days before the dawn of civilization. As the cave man emerged from the darkness into the light he felt insecure, hungry, lonely and inquisitive.

Maslow’s hierarchy of needs^2^, though accredited to him in the post-war era, is a mighty old doctrine. The instincts of mankind were well-known to the philosophers and thinkers of a thousand years old universities and seminaries of Qom, Najaf and Al - Azhar. Lessons in human psychology were taught as part of *falsafa* for hundreds of years.

Bergson an eminent French philospher talked about instinct and intellect. The entire animal kingdom has four common basic instincts i.e hunger, security, curiosity and companionship, albeit mankind possesses intellect, which makes it distinct and superior to the entire animal kingdom. No honour comes without a responsibility. No pain, no gain. So the intellect bestowed to mankind makes him liable for his actions. He is bound by a rope to a pillar of divine command, between free will and determinism. He has the power to differentiate Good from Evil. What he chooses is what leads to his rise or fall from the high altar.

Parents and teachers are fundamental pillars of a community. During the COVID pandemic it was not just the health personnel but the parents and teachers also who experienced Moral Trauma. Parents felt that they failed to provide such a quality of parenthood to their children to lock downs, isolation and damage to the natural growth of their children.

Human beings thrive on social interactions. Isolation led to loss of such interactions beyond the control of parents. So they felt squeamish, guilty and somehow responsible for loss of one whole year or more of the social development of their child. Like many other countries, educational institutions in Pakistan were also closed for almost a year and they have just opened. For some time they opted for online education but it cannot replace the utility of face to face education, hence their education has suffered a great deal. On the other hand, mental health issues and domestic abuse have been on the rise too.

Education means reformation of a personality. Knowledge enables the refinement of a personality in tangible palpable and visible forms. Informal education beginning at mother’s knee continues throughout one’s life where contact with friends, elders, peers, Murshad, gurus, teachers and mentors transmits knowledge and skills through osmosis. The formal education begins at the primary school and culminates at the University or an institute, etc. Both these forms are based upon direct interaction, better called face to face contact between people, and all that has lacked during the pandemic.

All states are cognizant of their responsibility and want to return to normal schooling as we have known for eons. But the nanoparticle continues to challenge the pride, pomp, and glory of mankind. For how long; only time will tell. *fatabiru ya ulil absar (* Quran 59:2) .”Learn a lesson, o beholder”.

If there is an illustration of Charles Dickens’ Bleak house in modern days, it has to be the present state of COVID affected mansions. Be it rich or poor, developed or underdeveloped, all went through the bleak nights of 2020. The emergence of vaccines through Pfizer bioNtech was like a fiery dawn at the end of a long tunnel. It gave the first glimmer of hope which was akin to an answer to a long wait for Goddot, in Samuel Beckett’s language.

West Yorkshire has thousands of Asian families who refused to accept the instructions by the health authorities mainly, social distancing, and continued to meet and greater in the summer and autumn of last years. Many hundreds suffered. Some survived to suffer with the long Covid, others, sadly, succumbed. Same happened to many Pakistani families in the US.

And yet, as many of us in health care know it too well, that our communities refuse to accept social distancing rules, vaccination and even the very existence of a pandemic. Black, Asian and Ethnic minorities (BAME) have suffered the most in the U.K. and denial of the pandemic and vaccines continues to dominate. Statement by health officials in Pakistan recently highlighting the side effects of vaccines and casting doubts on Chinese vaccine’s efficacy in elderly people over sixty years of age which were later clarified, should have been avoided in the first place. It did create some confusion with the result that the healthcare workers who were first offered vaccination with the result that healthcare workers in Islamabad has now adopted the policy of wait and see before getting themselves vaccined.^3^ So sad. Ignorance is indeed the worst enemy of mankind. A curse for humanity. However, in the last few days reports have appeared in the media that fourteen hundred vaccine doses at three government hospitals have gone missing or have been administered to unauthorized persons while many vials have been spoiled because of improper storage. There have been many reports on social media of celebrities and politicians' families getting vaccinated out of turn which must be checked.4 On the other hand, reluctance is still there in some sections of the society against vaccination for which the authorities need to create awareness about the benefits of vaccination against Covid19.

To conclude, the vaccine production, procurement, and availability has introduced a new debate called the Vaccine Nationalism, which must be condemned. All human beings are born free and equal. If the rich and the powerful start controlling and hegemonizing the availability of vaccines it resurfaces the same old doubts, namely what they preach they do not practice. What’s good for the goose should also be good for the gander. But not in the eyes of the high and the mighty.

Many moral and ethical debates have cropped up since the outbreak of the pandemic. One such debate is on the subject of moral injury, which is somewhat less known in the ethics circles. Take home message. Moral trauma caused by the COVID-19 has left indelible scars on the human psyche. They may remain permanently etched out on the memory cortex.
